# An ethnobotanical study of medicinal plants administered for the treatment of hypertension

**DOI:** 10.15171/jrip.2016.26

**Published:** 2016-08-09

**Authors:** Babak Baharvand-Ahmadi, Mahmoud Bahmani, Pegah Tajeddini, Mahmoud Rafieian-Kopaei, Nasrollah Naghdi

**Affiliations:** ^1^Madani Heart Hospital, Department of Cardiovascular, Faculty of Medicine, Lorestan University of Medical Sciences, Khorramabad, Iran; ^2^Clinical Microbiology Research Center, Ilam University of Medical Sciences, Ilam, Iran; ^3^Medical Plants Research Center, Shahrekord University of Medical sciences, Shahrekord, Iran

**Keywords:** Ethnobotanical, Hypertension, Medicinal plants

## Abstract

**Introduction:** The incidence of cardiovascular diseases (CVDs) is very high in human societies and their prevention and treatment are the most important priority in many countries. Hypertension makes an important contribution to the development of CVDs.

**Objectives:** This study aimed to collect the ethno-medicinal knowledge of the traditional healers of Shiraz on medicinal plants used in the treatment of hypertension.

**Materials and Methods:** Ethno-medicinal data were collected from September 2012 to July 2013 through direct interview. Twenty-five healers were interviewed using semi-structured questionnaires and their traditional ethno-medicinal knowledge was recorded. Questionnaires were included apothecary personal information, plant local name, plant parts used, method of preparation, season of harvest and traditional use. Data collected from surveys and interviews were transferred to Microsoft Excel 2007 and analyzed.

**Results:** Analysis of data showed that, 27 medicinal plants from 22 families are used for the treatment of hypertension. The families with most antihypertensive species were Apiaceae (8%), Rosaceae (8%) and Papaveraceae (8%). The most frequently used plant parts were leaves (36%) followed by fruits (30%), aerial part (17%) and branches (7%). The most frequently used preparation method was decoction (95%). *Borago officinalis* (51.85%), *Berberis vulgaris* (51.58%) had the highest frequency of mention.

**Conclusion:** The ethno-medicinal survey of medicinal plants recommended by traditional healers for the treatment of hypertension provides new areas of research on the antihypertensive effect of medicinal plants. In the case of safety and effectiveness, they can be refined and processed to produce natural drugs.

**Implication for health policy/practice/research/medical education:**

Hypertension known as high blood pressure which it is a long-term medical condition in which the blood pressure in the arteries is persistently elevated. Blood pressure is expressed by two measurements, the systolic and diastolic pressures, which are the maximum and minimum pressures, respectively, in the arterial system. In addition to chemical drugs that are commonly prescribed by physicians, there are various ways to comply with them to control the disease, such as exercise, quit smoking, reducing stress and use of medicinal plants in moderation. In our study showed that 27 medicinal plants from 22 families are used for the treatment of hypertension which could have the potential to produce natural remedies for high blood pressure.

## Introduction

The incidence of cardiovascular diseases (CVDs) is very high in human societies and their prevention and treatment are the most important priority in many countries. The use of medicinal plants in treatment and prevention of diseases have increased dramatically over the last years (1). High blood pressure can damage vessels of supplying blood to the heart, kidneys, brain, and eyes (2). It makes an important contribution to the development of coronary heart diseases, cardiovascular events and stroke (2,3). The overall death rate from hypertension is twice than that of the general population. Hypertension increases the risk of sudden death in patients with CVD. Smoking, hypercholesterolemia and diabetes are important risk factors for the development of hypertension (2).

Hypertension is a major threat to health, especially for older people. Over one-fourth of all death among the elderly people is due to high blood pressure and its complications. It is usually asymptomatic until the development of complications such as heart failure, stroke and kidney failure (3). High blood pressure is referred to as a silent killer (4). In 1997, there were on average 100 million physician office visits for hypertension among adults in the United States (5). Hypertension is the most important risk factor for the development of CVDs. CVDs are the largest cause of death in the developed countries and their prevalence are appears to be increasing in developing countries (6). There are 17 major causes of death in the world and cardiovascular is the seventh leading cause of death. In Iran, CVD is the first line cause of death (7). The cause of high blood pressure is unknown but several factors can contribute to the development of this condition. Obesity and raised body mass index increase the risks of high blood pressure (8). Scientific studies suggested an association between birth weight and blood pressure in children, adolescents and adults. Therefore, detection and control of hypertension is the primary objective for the prevention of CVDs (9).

Several simple methods can be used to control high blood pressure. It can be controlled with lifestyle changes, oral medications or both. So far several drug forms have been introduced to reduce and control high blood pressure, each of them has its own side effects (9). Use of herbal remedies is another way to treat and control hypertension. Medicinal plants are not only effective in the treatment of high blood pressure but also in many other situations (10). However, like other medications, they can lead to unwanted side effects or drug interactions, especially when taken in large doses (11). Vitamins found in vegetables, fruits and fish oil play an important role in the regulation of blood pressure. Thus, consumption of fish and fish oils, carrots, parsley, yellow chicory, apricot, tomato, lettuce, peas, dates and butter that are high in vitamin can be useful in treating hypertension (12). The current trend of medical world is toward using natural compounds in disease prevention and treatment. Medicinal plants, as the most precious gift of Allah to mankind, have long been used in Iranian folk medicine to treat various diseases. This thousand-year-old traditional medicine offers a practical and comprehensive guide for the application of medicinal plants and therefore, following its guidelines can be useful in resolving public health problem (13). Ethno-botanical and ethno-pharmacological surveys are studies intended to document traditional knowledge and use of medicinal plants. They also bring numerous ideas for pharmacological science (14). Recently, a great deal of attention has been focused on the finding of new drugs with minimal side effects and high compatibility with human nature (15). In addition to developing countries, in Western countries especially in Europe, trend toward traditional medicine has been growing despite access to modern medicine (16). Today, pharmaceutical science has made great advances, and herbal medicines are used beside chemical ones to treat various ailments (17). It has been reported that more than 30% of modern medicines are originally derived from medicinal plants (18). So far, a number of medicinal plants have been investigated for their therapeutic effects (19).

According to the high prevalence of hypertension in the world, especially in Iran and urgent need to discover new effective natural remedies this study aimed to document medicinal plants recommended by Shirazian herbalists for the treatment of hypertension.

## Objectives

This study aimed to collect the ethno-medicinal knowledge of the traditional healers of Shiraz on medicinal plants used in the treatment of hypertension.

## Materials and Methods

### Description of the study area

Shiraz is one of the major cities in Iran and the capital of Fars province. It is geographically located in the southwest of Iran and in the central of Fars province. The city of Shiraz is situated in the Zagros mountain range, 1468 m above the sea level and bounded by Drak mountain in the west and Sabzpushan, Bamu, Chelmagham and Babakuhi mountains in the North. Shiraz with a length of 40 km and a width of 15 to 30 km has a total area of 1268 square kilometers. Based on the 2009 census the total population of the city was about 1700678. Generally the city has a moderate climate. The hottest month in Shiraz is July, with an average temperature of 30 and the coldest month is January, with an average temperature of 5. The average annual temperature is about 18 and the average annual rainfall is 3378 mm.

### Ethical issues

The research followed the tenets of the Declaration of Helsinki. The research was approved by the ethical committee of Shahrekord University of Medical Sciences.

### Statistical analysis

The study was carried out from September 2012 to July 2013. Ethno-Medicinal data was collected from 27 traditional healers through face-to-face interviews. They were interviewed using semi-structured questionnaires and their traditional ethno-medicinal knowledge was recorded. Questionnaires were included apothecary personal information, plant local name, plant parts used, method of preparation, season of harvest and traditional use. Questionnaires data were then transferred to Microsoft Excel 2007 and processed.

## Results

**Figure 1:**
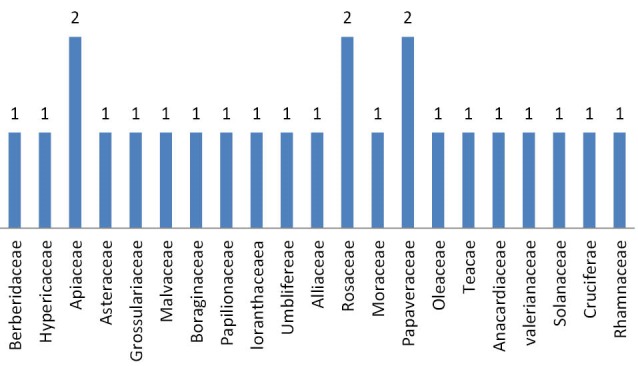
Plant families recommended by local healer for the treatment of hypertension.

**Figure 2:**
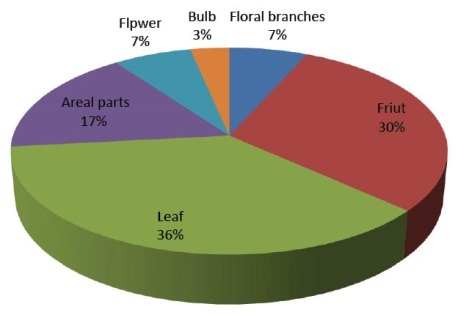
Percentage of different plant parts used to treat hypertension in the study area.

**Figure 3:**
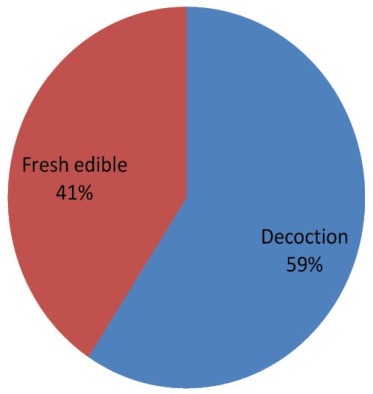
Percentage of different preparation methods of medicinal plants in the study area.

Ethno-medicinal information of plants recommended by Shirazian herbal healer are shown in Table 1. A total of 27 medicinal plant species belonging to 22 families were recommended by herbal healers for the treatment of hypertension. As shown in Table 2, *Borago officinalis* (51.85%), *Berberis vulgaris* (51.58%), *Anethum graveolens* (48.14%), *Coriandrum sativum* (44.44%), *Centaurea depressa M*. (44.44%), *Crataegus aronia* (44.44%), *Camellia sinensis* (44.44%), *Petroselinum sativum* (40.74%) and *Olea europaea* (40.74%) had the highest frequency of mention. Figure 1 shows plant families recommended by local healer for the treatment of hypertension. The families with most antihypertensive species were Apiaceae (8%), Rosaceae (8%) and Papaveraceae (8%). Analysis of data showed that leaves (36%) were the most frequently used plant parts followed by fruits (30%), aerial part (17%) and branches (7%, Figure 2). As shown in Figure 3, decoction (95%) was the most frequently used preparation method of medicinal plants.

**Table 1:** Medicinal plants used for the treatment of hypertension; scientific name, common name, family name, plant parts used and preparation methods

**Scientific name**	**Family**	**Persian names**	**Plant parts used**	**Preparation methods**
*Centaurea depressa M.*	Compositae	Golegandom	Seed	Decoction
*Berberis vulgaris*	Berberidaceae	Zereshk	Fruit	Decoction
*Hypericum perforatum*	Hypericaceae	Chay-Koohi	Leave	Decoction
*Anethum graveolens dhi*	Apiaceae	Shevid	Leave	Fresh
*Coriandrum sativum*	Apiaceae	Geshniz	Leave	Fresh
*Cichorium intybus L*	Asteraceae	Kasni	Leave	Decoction
*Ribes divaricatum*	Grossulariaceae	Angoor	And Fruit‏ Leave	Fresh
*Althea aucheri Boiss.*	Malvaceae	Khatmi-Armanestani	Aerial parts	Decoction
*Borago officinalis*	Boraginaceae	Gavzaban	Fruit	Decoction
*Gundelia tournefortii L.*	Compositae	Kangar	Leave	Fresh
*Trigonella monspeliaca*	Papilionaceae	Shanbalileh-Monileei	Leave and Fruit‏	Fresh
*Viscum album*	Loranthaceae	Darvash	Aerial parts	Decoction
*Petroselinum sativum*	Umbelliferae	Jafari	Leave	Fresh
*Allium sativum*	Alliaceae	Sir	Bulb	Fresh
*Crataegus aronia*	Rosaceae	Zalzalak	Fruit	Fresh
*Ficus religiosa*	Moraceae	Anjir	Fruit	Fresh
*Glaucium oxylobum Boiss & Buhse*	Papaveraceae	Shaghayegh-Goltiz	Leave	Decoction
*Glaucium grandiflorum Boiss & Huet.*	Papaveraceae	Shaghayegh-Goldorosht	Leave	Decoction
*Olea europaea*	Oleaceae	Zeytoon	Leave and Fruit	Decoction
*Camellia sinensis*	Teacae	Chay-sabz	Leave	Decoction
*Rhus Coriaria. L*	Anacardiaceae	Somagh	Fruit	Decoction
*Matricaria recutita*	Asteraceae	Babooneh	Flower	Decoction
*Valeriana officinalis*	valerianaceae	Sonboletib	Aerial parts	Decoction
*Cotoneaster persica Pojark.*	Rosaceae	Shirkhest	Aerial parts	Decoction
*Physalis alkekengi*	Solanaceae	Aroosak-Poshtpardeh	Aerial parts	Decoction
*Descurainia Sophia (L.) Schr.*	Cruciferae	Khakshir Irani	Fruit	Fresh
*Ziziphus zizyphus*	Rhamnaceae	Annab	Fruit	Fresh

**Table 2:** Frequency of mentions of anti-hypertensive effects for each plant spices

**Scientific name**	**Persian name**	**The number of herbalists mentioned the plant**	**Frequency of citation (FC) percentage (%)**
*Centaurea depressa M.*	Golegandom	12	44.44
*Berberis vulgaris*	Zereshk	14	51.58
*Hypericum perforatum*	Chayekohi	6	22.22
*Anethum graveolens dhi*	Shevid	13	48.14
*Coriandrum sativum*	Geshniz	12	44.44
*Cichorium intybus L*	Casni	10	37.03
*Ribes divaricatum*	Angoor	13	48.14
*Althea aucheri Boiss.*	Khatmi-Armanestani	9	33.33
*Borago officinalis*	Gavzaban	14	51.85
*Gundelia tournefortii L.*	Kangar	5	18.51
*Trigonella monpeliaca*	Shanbalileh-Monilileei	7	25.92
*Viscum album*	Darvash	5	18.51
*Petroselinum sativum*	Jafari	11	40.74
*Allium sativum*	Sir	10	37.03
*Crataegus aronia*	Zalzalak	12	44.44
*Ficus religiosa*	Anjir	3	11.11
*Glaucium oxylobum Boiss & Buhse*	Shaghayegh-Labtiz	1	3.70
*Glaucium grandiflorum Boiss & Huet.*	Shaghayegh-Goldorosht	2	7.40
*Olea europaea*	Zeytoon	11	40.74
*Camellia sinensis*	Chay-Sabz	12	44.44
*Rhus Coriaria . L*	Somagh	4	14.81
*Matricaria recutita*	Babooneh	5	18.51
*Valeriana officinalis*	Sonboletib	5	5
*Cotoneaster persica Pojark.*	Shirkhesht	4	14.81
*Physalis alkekengi*	Aroosak-poshtpardeh	4	14.81
*Descurainia Sophia (L.) Schr.*	Khakshir-Irani	3	3
*Ziziphus zizyphus*	Annab	1	3.70

Figures 1, 2 and3, respectively show the plant families, Percentage of different plant parts used, and percentage of different preparation methods of medicinal plants for anti-hypertensive plants in the study area.

## Discussion

In this study, we collected ethno-medicinal knowledge from herbal healer in Shiraz for the use of medicinal plants as a treatment for hypertension. A total of 27 medicinal plant species belonging to 22 families were documented in the present study for the treatment of hypertension. A wide variety of medicinal plants are used to treat hypertension in different parts of Iran. *Rumex crispus L* and *Ziziphus jujuba (L) H. Karst* and *Olea europaea* L are traditionally used in Mobarakeh of Isfahan (20). In Arasbaran region (Northwestern Iran), *Berberis vulgaris L., Achillea millefolium L., Ecballium elaterium, Ribes orientale, Crataegus monogyna*, and *Taxus baccata L* are used to treat high blood pressure (21). In Sistan and Baluchestan province, Nigella (*Nigella sativa* L.) is used for this purpose (22). *Silybum marianum (L.) Gaerth, Achillea tenuifolia, Cichorium intybus, Silybum marianum, Berberis vulgaris, Capsella bursa-pastoris, Equisetum arvense, Juglans regia,* and *Melilotus indicus* are used in Kazerun (Southern Iran) to treat hypertension (23). In Lorestan province, *Falcaria vulgaris, Smyrnium cordifolium, Crocus haussknechtii, Berberis integerrima, Ziziphus spina-christi, Ziziphus nummularia, Allium ursinum, Tragopogon porrifolius, Anethum graveolens* are thought to have blood pressure lowering effect (24). *Rheum ribes L.* and *Paliurus spina-christi* are used to lower blood pressure in Ilam province (25). Comparison of medicinal plants used for treating hypertension in different parts of Iran shows that different cultures and cities of Iran use different plants to treat hypertension however, some common plants are also used in different area. In this study, several plant species were documented for the first time to treat hypertension and may have the potential to produce antihypertensive drugs.

The exact mechanisms responsible for the anti-hypertensive effect of medicinal plants are not fully understood and needed to be investigated. As mentioned, several factors and conditions can lead to increased blood pressure level (12). Several findings suggest that increased oxidative stress potentially contribute to the elevation of blood pressure level (6). Antioxidant activity is one of the most important biological effects of medicinal plants and this effect is mainly attributed to the presence of secondary metabolites, especially phenolic compounds. Therefore, the hypertensive effect of medicinal plans may be related to the attenuation oxidative stress. Thus, it can be said that other medicinal plants with antioxidant effects may be effective in the treatment of hypertension.

## Conclusion

The ethno-medicinal survey of medicinal plants recommended by traditional healers for the treatment of hypertension provides new areas of research on the antihypertensive effect of medicinal plants. In the case of safety and effectiveness, they can be refined and processed to produce natural drugs.

## Limitations of the study

This study was limited to a part of Iran. The same study in various parts of Iran is suggested.

## Acknowledgments

The authors accomplish this research by the support of Shahrekord University of Medical Sciences, Shahrekord, Iran (Grant# 9/7995).

## Authors’ contribution

All the authors wrote the first draft of the manuscript equally. MRK revised and edited the last version.

Authors declare no conflict of interests.

## Conflicts of interest

Authors declare no conflict of interests.

## Ethical consideration

Ethical issues (including plagiarism, data fabrication, double publication) have been completely observed by authors.

## Funding/Support

None.
